# Sourdough bread as nutritional intervention tool for improvement of cognitive dysfunction in diabetic rats

**DOI:** 10.1186/s40795-024-00861-x

**Published:** 2024-03-26

**Authors:** Doha A. Mohamed, Karem Fouda, Hoda B. Mabrok, Marwa E. El-Shamarka, Ibrahim M. Hamed

**Affiliations:** 1 Nutrition and Food Science Department, Food Industries and Nutrition Institute, National Research Centre, Dokki, Cairo, 12622 Egypt; 2 Toxicology and Narcotics Department, Medical Research and Clinical Studies Institute, National Research Centre, Dokki, Cairo, 12622 Egypt

**Keywords:** Sourdough bread, Streptozotocin-Nicotinamide, Type-2 diabetes, Gene expression, Biochemical parameters, Cognitive function

## Abstract

**Background:**

The current research targeted to study the impact of nutritional intervention by two sourdough breads in improvement of cognitive dysfunction in diabetic rats.

**Methods:**

Type-2 diabetes was induced in rats by Streptozotocin-Nicotinamide (STZ-NC). Diabetic rats were fed on balanced diet or balanced diet containing 20% of sourdough bread I or II for a month. Lipid profile, oxidative stress, inflammatory markers and cognitive functions were assessed in all rats. Gene expression of brain-derived neurotrophic factor (BDNF) and nuclear respiratory factor 2 (NRF-2) were assessed in hippocampal tissue, while expression of phosphoenol pyruvate carboxy kinase (PEPCK), and glucose transporter 2 (GLUT2) genes were evaluated in hepatic tissue. Chemical composition and fatty acids profile were evaluated in the prepared sourdough bread.

**Results:**

Sourdough bread II showed higher content of phenolic compounds, fat, fiber and carbohydrates. Fatty acids profile revealed that sourdough bread I was higher in saturated fatty acids (16.08%), while sourdough bread sample II was higher in unsaturated fatty acids (79.33%). Sourdough bread I or II feeding rats’ showed significant improvement in hyperglycemia, oxidative stress markers, inflammatory markers, lipid profile, liver and kidney functions in association with improvement in cognitive function. Gene expression of BDNF and NRF2 in hippocampal tissue were increased significantly, while hepatic GLUT2 and PEPCK gene expression were down-regulated in diabetic given sourdough bread I or II.

**Conclusion:**

Sourdough bread II was superior in all the studied parameters. The anti-diabetic effect and protection from cognitive dysfunction of sourdough bread samples may be ascribed to the occurrence of dietary fibers, phenolic compounds, and polyunsaturated fatty acids.

## Background

Diabetes mellitus is considered as one of the non-communicable disease affecting globally 537 million of people and this number rise to 783 million by 2045 [1]. People older than 65 years accounted about 19% of diabetic patients, while diabetic patients ranged from 65 to 99 years estimated 123 million globally [2]. The prevalence of Alzheimer’s disease (AD) is elevated in diabetic patients (65%) than normal subjects [3]. Type2-diabetes (T2D) causes brain oxidative stress, insulin resistance, and cognitive dysfunction [2, 4]. Glucose intolerance, insulin resistance and impaired insulin excretion are the main linkage between T2D and AD [5]. Due to the conjunction of the reasons for T2D and AD a new name is created, which is Type 3 diabetes referring to AD as form of AD diabetes that especially involve the brain [6]. According to World Health Organization (WHO) T2D is consider as risk factor for dementia [7]. Consequently, diabetes and its disorders must be controlled for AD prevention [5]. Brain-derived neurotrophic factor (BDNF) is a neurotrophic factor act as neurotransmitter mediator, stimulates neuron regeneration, connection, decrease liver gluconeogenesis and promote liver insulin signal transduction [8]. Nuclear factor erythroid 2-related factor 2 (Nrf2) regulate BDNF, suppressed oxidative stress, decrease expression of genes related to gluconeogenesis such as GLUT2 and increase cellular glucose uptake [9]. Injection of insulin as treatment for diabetes induces neurotrophic factors (BDNF) in hippocampus which has an improving effect on memory performance of Morris water maze test [10]. Insulin impact on cognition may be mediated through glucose metabolism regulation in cortex and hippocampus region which responsible for learning and memory in brain. This action is mediated by elevation in glucose transporter genes (GLUTs) and glucose uptake [11]. Shi et al. [12] reported that a natural food additive called trilobatin has anti-diabetic effect through activation of IRD-1/GLUT2 pathway and NRF2/ARE pathway. Hence, producing a novel food product has an effect on glucose transporter signaling as well as NRF2 signaling might serve as a promising candidate for treatment of diabetic and prevent from cognition dysfunction.

Conventional sourdough starter is an old dough fermenting factor made by blending flour and water, put up with spontaneous fermentation through yeast and lactic acid bacteria (LAB). Sourdough enhance bread quality by prolongation of shelf life, augmented loaf volume, retard staling, enhance sensory characteristics, and increased nutritional value [13–15]. Conventional sourdough breads have proven health beneficial effects such as lowering of risks related to colorectal cancer, obesity, cardiovascular diseases and diabetes. The sourdoughs used in bread production usually contain a wide range of probiotics which give more beneficial activities towards human health [16]. Sourdough fermentation has been shown reduction in glycaemic index of baked products and enhances phytochemical content and increases the availability of minerals through reduction of anti-nutritional factors [17]. Fibers, polyphenols and polyunsaturated fatty acids have prebiotic properties and play an important role in improving cognitive performance in animal models through influence on biochemical pathways such as brain derived neurotropic factor [18, 19]. Thus, nutritional intervention by fermented foods in form of sourdough bread containing plant food ingredients rich in phytochemicals (phenolic compounds, polyphenols and flavonoids) and phytonutrients (polyunsaturated fatty acids and fibers) may be a good strategy in treatment of type 2 diabetes and its complication in cognitive function impairment. The goal of the current investigation was preparation of two sourdough bread formulae and evaluation of their hypoglycaemic effect in type-2 diabetes induced in rats by Streptozotocin/Nicotinamide (STZ/NC). The impact of diabetes in biochemical parameters, molecular and behavioural parameters were assessed in all rats. Chemical analysis and fatty acids profile of the prepared sourdough bread were evaluated.

## Methods

### Plant materials

All material were purchased from local market (Cairo, Egypt) including sesame seeds, quinoa seeds, chickpea seeds, lupin seeds, whole wheat flour, wheat germ, oat, sunflower oil, sesame oil, yeast, salt and yoghurt.

### Animals

Male rats of Sprague Dawley of average body weight 154.4 g were used in the current research. Animals were purchased from the National Research Centre Animal house. During the study, the animals were given clean water and normal laboratory feeds *ad-libitum*. All animals were housed individually in cages. The animal procedures were done according to our institutional research and ethics committee and in line with the ethical guideline for animal care and use for scientific purposes developed by the National Research Centre, Egypt (19176).

### Animal’s diets

Three types of diet were designed and fed to animals during the entire study term (Table 1). AIN-93 vitamin and salt mixture were prepared according to Reeves et al. [20]. The prepared two sourdough bread samples were dehydrated and crush into powder prior their addition to the diet.

**Table 1:** Composition of different experimental diets (g per 100 g)

**Ingredients**	**Balanced diet**	**Sourdough bread I diet (**DBI**)**	**Sourdough bread II diet (**DBII**)**
^a^**Casein**	12	8.31	8.31
**Corn oil**	10	5.83	7.9
**Sucrose**	22.8	22.8	22.8
**Starch**	45.7	33.56	31.49
**Salt mix.**	3.5	3.5	3.5
**Vitamin mix.**	1	1	1
**Cellulose**	5	5	5
**Sourdough bread I powder**	-	20	-
**Sourdough bread II powder**	-	-	20

^a^12 g casein has been determined to contain 10 g protein using AOAC[21]

### Methods

#### Preparation of sourdough

Liquid sourdough starter (LTSS) was prepared through fermentation of whole wheat flour and water in equal ratios according to the methods of Rózyło et al. [22] and Couch [23]. Twenty –four hour after incubation at 34 °C, of the preferment’s was refreshed each 7h/day with whole wheat flour and water till mature. Moreover, the mature sourdough starter was identified through the emergence of bubbles, the evanescence of the rotten milk smell, the emergence of a sweet smell, and a doubling in volume. So, sourdough was readiness for using in bread preparation.

#### Preparation, proximate analysis, phenolic compounds content and fatty acids profile of sourdough bread samples

Two sourdough (I and II) were intended. Bread I ingredients were sesame seeds, quinoa seeds, chickpea seeds (soaked, boiled and dried), sesame oil, yeast, yoghurt, salt and flour of whole wheat. Bread II was consisted of lupin seeds (soaked, boiled and dried), oat, wheat germ, sunflower seeds, yeast, yoghurt, salt and flour of whole wheat.

Sourdough was mixed with the dried ingredients of powder mixture of bread I and II in a spiral type KM400 dough mixer (Kenwood Havant, Hampshire, UK) for 10 min, temperature of dough after mixing was 25 ℃. Dough was incubated for 30 min at 35 ◦C. Bread samples were baked in oven for 45 min at 200 ± 10 ℃ after 30 min of incubation. After cooling to ambient temperature, proximate composition of bread samples were analyzed [21]. Folin-Ciocalteu reagent was used in the estimation of total phenolic content and the results were represented as gallic acid equivalents (GAE) in mg/100g sample[24]. The results were expressed as mean ± SD for three replicates. Fatty acids methyl esters were prepared according to AOAC [21] was used for preparation of methyl esters of fatty acids of the formulated sourdough bread for GLC analysis of fatty acids. Fatty acids methyl ester were identified and estimated of the executed using the condition utilized in Moha––med et al. [25].

#### Induction of experimental diabetes

Freshly prepared streptozotocin (STZ) (60 mg/kg BW) were intraperitoneally (ip) injected in rats after 15 min from nicotinamide (NA) (ip 110 mg/kg BW) administration [26]. Fasting blood glucose was measured after 72 h after injection of STZ-NA for proven of T2D in rats (values above 200 mg/dl).

#### Study protocol

Twenty-four rats were split into four groups (6 rats in group) as follows:

Group1: Normal control rats, Group 2: diabetic control rats (DC), Group 3: diabetic rats fed on balanced diet containing 20% sourdough bread I and Group (4) diabetic rats fed on balanced diet containing 20% sourdough bread II. Rats in groups one and two were rats fed on balanced diet all the experimental period. The impact of diabetes on cognitive functions was assessed in rats through behavioural assessment (MWM, Y-maze, and new object recognition). Throughout the research, food intake and body weight were observed weekly. After five weeks all nutritional parameters (total food intake, body weight gain and feed efficiency ratio) were evaluated.

#### Rat’s anesthesia for collection of plasma and tissues samples

After overnight fasting, rats were anesthetized with peritoneal injection of 6.6 mg/kg Ketamin and 0.3 mg/ kg of Xylazine. Cardiac puncture blood samples were obtained and centrifuged for plasma extraction. Plasma were detached from all blood samples for estimation of blood glucose [27], insulin (ELISA kit, Catalogue # SL0373Ra Sunlong**®**), malondialdehyde (MDA) [28], catalase [29], C-reactive protein (CRP) (ELISA kit, Catalogue # SL0202Ra Sunlong**®**), tumour necrosis factor-α (TNF-α) (ELISA kit, Catalogue # SL0722Ra, Sunlong**®**). Functions of liver (AST and ALT) and kidney (urea and creatinine) were evaluated using colorimetric kits. Plasma lipid profile was determined (total cholesterol, triglycerides, HDL-cholesterol and LDL-cholesterol) using colorimetric kits. Ratios of T-Ch/HDL-Ch and TGs/HDL-Ch were calculated. Functions of liver (AST and ALT) and kidney (urea and creatinine) were evaluated using colorimetric kits. Plasma lipid profile was determined (total cholesterol, triglycerides, HDL-cholesterol and LDL-cholesterol) using colorimetric kits. Ratios of T-Ch/HDL-Ch and TGs/HDL-Ch were calculated. Consequently, the rats were sacrificed by decapitation under guillotine consisting of a metal frame and a sharp blade, and being operated by one hand. After decapitation of animals, the liver and brain samples were dissected for gene expression analysis.

#### Gene expression of BDNF, NRF2, GLUT2 and PEPCK

PureLink®RNA Mini kit (Ambion®Life-technologies^TM^) was used to isolate total RNA from frozen hippocampus and liver tissue according to the manufacturer’s guidelines. Total RNA levels and RNA purity were measured using Nanodrop-spectrophotometer. RevertAid first strand cDNA synthesis kit (ThermoFisher® invitrogen^TM^) was used to synthesize cDNA from RNA (1.5µg) according to the manufacturer’s guidelines.

Real-time PCR was performed with a Rotor-Gene® MDx instrument. The reaction mixture of 25 µl was contained cDNA (1 µl), master mix (SYBR-Green®PCR master mix, ThermoFisher® invitrogen^TM^) and primer pairs (0.25 µM). The sequence of primer pairs used for Brain-derived neurotrophic factor (BDNF) (Gene ID: 116901726), Nuclear respiratory factor 2 (NRF-2) (Gene ID: 116901536), Phosphoenol pyruvate carboxy kinase (PEPCK) (Gene ID:116902279), and Glucose transporter 2 (GLUT2) (Gene ID:116896985) genes expression determination were presented in Table 2. The protocol for the reaction of PCR used as follow: 2 min at 50°C thereafter 10 min at 95°C subsequently 45 cycles consist of 20 Sec at 95°C, 60 Sec at 60°C, 30 Sec at 72°C then melting curve programme. The target genes relative expressions were calculated using the method of delta CT [30] and it was normalized to the expression of the house-keeping-gene glyceraldhyde-3-phosthate dehydrogenase (GAPDH) (Gene ID:116884019).

**Table 2:** The Sequence of primer used for RT-PCR analysis

**Target genes**	**Sequences**	**Ref**
**NRF2**	FW (5′-*CACATCCAGACAGACACCAGT*-3′) RW(*CTACAAATGGGAATGTCTCTGC*-3′)	[31]
**BDNF**	FW (5′-GTTGCATGAAGGCTGCGCCC -3′) RW(5′-CTGCCCTGGGCCCATTCACG-3′)	[32]
**GLUT2**	FW (5′-CTGGGTCTGCAATTTCATCA-3′) RW(5′-CGTAAGGCCCGAGGAAGT-3′)	[33]
**PEPCK**	FW (5′-*GATGACATTGCCTGGATGAA-3*′) RW(5′-*AACCGTTTTCTGGGTTGATG*-3′)	[33]
**GAPDH**	FW (5′- GTATTGGGCGCCTGGTCACC -3′) RW(5′- CGCTCCTGGAAGATGGTGATGG -3′)	[34]

#### The impact of diabetes on cognitive functions (behavioural assessment)

Three behavioural tests were evaluated in the diabetic rats. These tests were y-maze test, which estimates short memory [35] , Morris water maze (MWM), which assesses the learning capacity and visuo-spatial memory of animals [36] and the object recognition test, which test long-term memory (LTM) and evaluate cognition[37]. All behavioural assessment tests were carried according to the methods of Mohamed et al. [38].

### Statistical analysis

SPSS v26 statistical programme was used to analyse the data, one-way ANOVA with Tukey multiple comparison test was performed to evaluate whether the means are statistically different. The results of bread samples analysis were statistical analysed using Student’s t-test. Normal distributions of values were test by Kolmogorov-Smirnov normality test. Differences were considered significant at p≤0.05.

## Results

Table 3 represented the chemical composition and fatty acids profile of the two sourdough bread samples. The results revealed that both bread samples I and II contain similar content of protein (13.24% and 13.14%, respectively) and ash (1.75% and 1.76%, respectively). Sourdough bread I showed higher content of fat (13.97%) compared to sourdough bread II (7.49%), while sourdough bread II presented high fiber (5.66%) and carbohydrates (71.96%) content compared to sourdough bread I. Total phenolic compounds were presented in sourdough bread I and II by 636.4 and 976 mg GAE/100g sample. GC analysis of fatty acids methyl esters of the studied bread samples revealed that sourdough bread I and II contain palmitic acid was the main saturated fatty acid presents in both samples and appeared by 16.08% and 11.65%, respectively. Linoleic acid ω-6 (38.14%) was the major unsaturated fatty acid present in sourdough bread I, while linolenic acid ω-3 (39.71%) was the major unsaturated fatty acid present in sourdough bread II. Oleic acid was present in sourdough bread I and II by 15.82% and 17.87%, respectively. Sourdough bread I (16.08%) was higher in saturated fatty acids compared with sourdough bread II (11.65%), while sourdough bread II (79.33%) was higher in unsaturated fatty acids compared with sourdough bread I (53.65%).

**Table 3:** Chemical composition and fatty acids profile of sourdough bread samples (Mean±SD)

**Parameters**	**Sourdough bread I**	**Sourdough bread II**
**Protein (g/100g)**	13.24^a^±0.041	13.14^a^±0.053
**Fat (g/100g)**	13.97^a^±0.094	7.49^b^±0.086
**Ash (g/100g)**	1.75^a^±0.041	1.76^a^±0.065
**Fiber (g/100g)**	4.84^a^±0.069	5.66^b^±0.114
**Total carbohydrates (g/100g)**	66.20^a^±0.241	71.96^b^±0.318
**Total phenolic (mg GAE/100 g)**	636.40^a^±3.332	976.0^b^±5.532
**Fatty acids (as percentage of total fatty acids)**		
**Lauric acid: C12 (0)**	0.466±0.003	-
**Myristic acid: C14 (0)**	1.044±0.019	-
**Plamitic acid: C16 (0)**	14.57^a^±0.102	11.65^b^±0.041
**Oleic acid: C18 (1)**	15.82^a^±0.089	17.84^b^±0.045
**Linoleic acid: C18(2)**	38.14^a^±0.123	21.78^b^±0.061
**Linolenic acid:C18 (3)**	4.69^a^±0.102	39.71^b^±0.078
**Total identified saturated fatty acids**	16.08^a^±0.111	11.65^b^±0.041
**Total identified unsaturated fatty acids**	58.65^a^±0.212	79.33^b^±0.161

Similar letters mean non-significant difference within groups (*p*≤0.05) in the same raw

Table 4 showed plasma levels of glucose, insulin, oxidative stress and inflammatory markers of different experimental groups. The results revealed that plasma levels of glucose were elevated (163.4%) significantly in diabetic control rats compared with normal. Plasma levels of insulin were reduced significantly in diabetic control rats compared with normal by 39.6%. Elevation of plasma glucose and reduction of plasma insulin in diabetic control rats are indicator to success of STZ as a model in induction of diabetes in rats. Rats feeding on balanced diet containing sourdough bread I or II noticed significant reduction in plasma glucose levels compared with diabetic control by 56.8% and 58.2%, respectively. Rats’ groups feeding on balanced diet containing sourdough bread I or II recorded significant elevation in plasma levels of insulin by 43% and 56%, respectively. Plasma MDA as indicator of lipid peroxidation was elevated significantly in diabetic control (118.6%) rats compared with normal rats, while plasma catalase as indicator to antioxidant status reduced significantly in diabetic rats (51.1%) in comparison to normal control rats. Elevation in plasma MDA in association with reduction of catalase is indicator to elevation of oxidative stress status in diabetic rats. Plasma TNF-α and CRP as inflammatory markers recorded significant elevation in diabetic control when compared with normal rats by 114% and 100%, respectively. Diabetic rats feeding on balanced diet containing sourdough bread I or II showed significant reduction in plasma levels of oxidative stress (MDA & catalase) and inflammatory markers (TNF-α and CRP) with different degrees.

**Table 4:** Plasma glucose, insulin, oxidative stress markers and inflammatory markers of different groups

**Parameters**	**Normal control**	**Diabetic control**	**Sourdough bread I**	**Sourdough bread II**
**Glucose (mg/dl)**	79.01^a^±2.32	208.1^b^±7.1	89.96^a^±2.31	87.03^a^±2.43
**Insulin (mU/l)**	6.47^c^±0.15	3.91^a^±0.09	5.89^b^±0.07	6.10^b^±0.09
**Oxidative stress markers**				
**MDA (nmol/ml)**	7.32^a^±0.36	16.00^d^±0.53	14.00^c^±0.53	12.62^b^±0.53
**Catalase (U/l)**	420.82^d^±3.35	205.62^a^±4.14	380.62^b^±6.21	397.50^c^±5.00
**Inflammatory markers**				
**TNF**-α** (pg/ml)**	13.90^a^±0.22	29.75^c^±0.89	21.50^b^±0.58	21.25^b^±0.64
**CRP (ng/ml)**	2.65^a^±0.12	5.30^d^±0.15	4.32^c^±0.08	3.67^b^±0.10

Similar letters mean non-significant difference within groups (*p*≤0.05) in the same raw

Table 5 showed plasma lipid profile, liver and kidney functions of normal and different diabetic rats groups. The results revealed that diabetic control group showed significant elevation in plasma levels of total cholesterol (78.5%), triglycerides (69.9%), LDL-cholesterol (218.3%) in association with reduction of HDL-cholesterol levels (22.6%) when compared with normal rats. The atherogenic ratio total cholesterol/HDL-cholesterol elevated significantly by 129.5% in the rats of diabetic control. Noteworthy raise were witnessed in liver (AST and ALT) and kidney (urea and creatinine) functions of diabetic control rats compared with normal rats. Feeding diabetic rats on balanced diet containing sourdough bread I or II improved plasma lipid profile with different degrees. AST and ALT as indicator to liver function were retarded to normal levels in rats feeding on balanced diet containing sourdough bread I or II. Also plasma urea and creatinine as kidney function indicator recorded significant reduction with different degrees compared with diabetic control in rats’ groups feeding on balanced diet containing sourdough bread I or II.

**Table 5:** Plasma lipid profile, liver and kidney functions of different experimental groups

**Parameters**	**Normal control**	**Diabetic control**	**Sourdough bread I**	**Sourdough bread II**
**T. Cholesterol (mg/dl)**	91.26^a^±1.80	162.86^c^±8.10	120.32^b^±2.17	110.31^b^±2.00
**TG (mg/dl)**	88.00^a^±1.93	149.50^c^±4.75	112.80^b^±3.88	100.80^ab^±6.27
**HDL-Ch (mg/dl)**	42.13^b^±0.64	32.61^a^±0.48	46.05^c^±1.84	44.75^bc^±0.94
**Cholesterol/HDL**	2.17^a^±0.06	4.98^c^±0.21	2.64^b^±0.12	2.47^ab^±0.06
**LDL-Ch (mg/dl)**	31.54^a^±2.04	100.40^c^±7.95	51.71^b^±2.34	45.40^b^±2.05
**TG/HDL**	2.09^a^±0.06	4.59^b^±0.15	2.48^a^±0.14	2.26^a^±0.15
**Urea (mg/dl)**	24.47^a^±1.43	32.72^b^±1.18	27.80^a^±1.23	25.26^a^±2.08
**Creatinine (mg/dl)**	0.43^a^±0.03	0.67^b^±0.09	0.53^ab^±0.03	0.43^a^±0.05
**AST (IU/l)**	31.50^a^±0.36	44.25^b^±4.21	32.75^a^±1.71	32.50^a^±0.84
**ALT (IU/l)**	23.32^a^±0.81	32.50^c^±1.05	26.50^b^±0.73	25.00^ab^±1.19

Similar letters mean non-significant difference within groups (*p*≤0.05) in the same raw

Table 6 indicated parameters of nutritional evaluation of different investigational groups. Rats of diabetic control revealed meaningful decrease in the gain of body weight and the ratio of food efficiency compared with all the studied rats groups, while the remained nutritional parameters and relative liver weight showed non-significant changes. The gain of body weight, final weight of the body, and liver relative weight of rats feeding on balanced diet containing sourdough bread I or II showed non-significant changes compared with normal rats group.

**Table 6:** Nutritional parameters and relative liver weight of different experimental groups

**Parameters**	**Normal control**	**Diabetic control**	**Sourdough bread I**	**Sourdough bread II**
**Initial body weight (g)**	145.50^a^±5.24	145.37^a^±6.03	145.37^a^±5.04	145.25^a^±4.74
**Final body weight (g)**	222.82^a^±6.45	207.25^a^±4.84	220.00^a^±5.55	222.00^a^±5.42
**Body weight gain (g)**	77.32^b^±1.25	61.87^a^±2.92	74.62^b^±3.28	76.75^b^±1.87
**Total food intake (g)**	589.17^a^±1.56	607.12^b^±1.52	593.25^ab^±6.48	595.75^ab^±6.88
**Food efficiency ratio**	0.13^b^±0.002	0.10^a^±0.005	0.13^b^±0.005	0.13^b^±0.004
**Liver weight (g)**	5.51^a^±0.18	5.99^a^±0.23	6.46^a^±0.37	6.30^a^±0.46
**Relative liver weight**	2.48^a^±0.06	2.91^a^±0.14	2.96^a^±0.21	2.87^a^±0.27

Similar letters mean non-significant difference within groups (*p*≤0.05) in the same raw

**Fig. 1:**
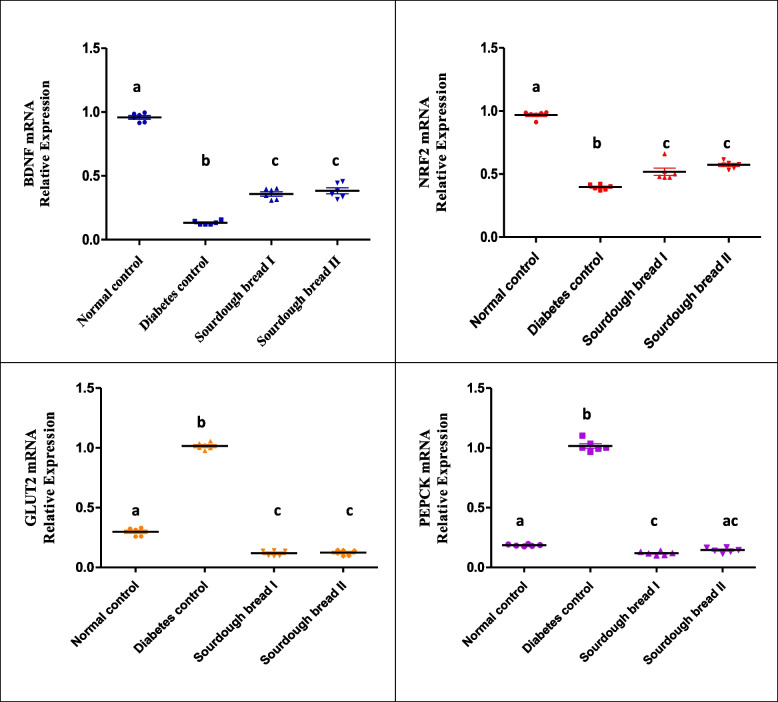
The mRNA gene expression of BDNF and NRF2 in hippocampal tissue and GLUT2 and PEPCK in liver tissue of different experimental rats. The mRNA expression is normalized with housekeeping gene (GAPDH), values are representing as means± SE. Similar letters mean non-significant difference within groups at *P*≤0.05

The mRNA gene expression of BDNF and NRF2 in hippocampal tissue (Fig. 1) were significantly (*p*< 0.001) down-regulated in diabetic control rats compared to normal control rats by 86% and 58%, respectively. Feeding on balanced diet containing sourdough bread I and II significantly increased the levels of BDNF (*p*< 0.01) gene expression by 2.6 and 2.9 fold changes, respectively. The mRNA levels of NRF2 were significantly elevated by sourdough bread I and II by 29 and 32%, respectively.

Hepatic GLUT2 and PEPCK gene expression (Fig. 1) were significantly (*p*< 0.001) up-regulated in rats of diabetic-control in comparison with rats of normal group (Fig. 1). Feeding on balanced diet containing sourdough bread I and II significantly (*p*< 0.001) decreased the levels of GLUT2 and PEPCK gene expression. Sourdough bread I and II I and II down-regulated GLUT2 gene expression by 8.5 and 8 fold-change, respectively. Sourdough bread I and II down-regulated PEPCK gene expression by 8.5 and 7 fold-change, respectively.

### Behavioural studies

The impact of diabetes in cognitive functions was evaluated through studying the different behavioural tests of all diabetic rats compared to normal rats. Diabetes leads to cognitive dysfunction as clarified in the investigated behavioural tests (water maze test, Y maze test and novel object recognition).

### Y–maze test

**Fig. 2:**
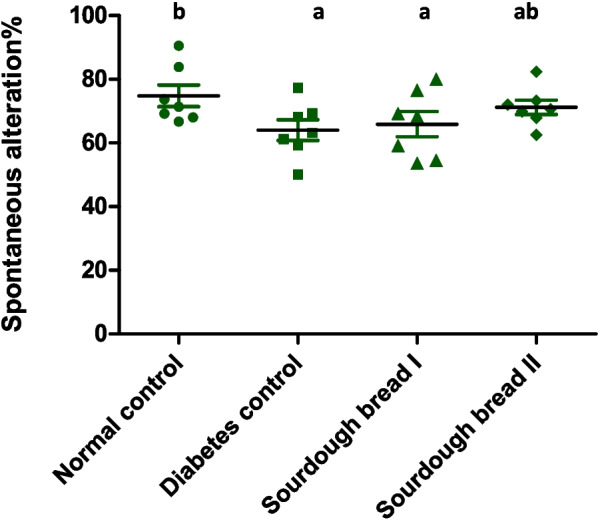
Y-maze test of normal and diabetic groups. Similar letters mean non-significant difference within groups at *P*≤0.05

Short-term memory is evaluated by Y-maze test. Diabetes leads to significant decreased in the percentage change seen in the diabetic control by 10.75% (Fig. 2) from those noticed in the normal rats. Feeding on balanced diet containing sourdough bread II showed significant elevation in percentage change in comparison to the diabetic control, while diet containing sourdough bread I showed non-significant elevation compared with diabetic control. However, the changes were still lower than that witnessed in the normal control.

### The Morris water maze test (MWM)

The MWM is applied to reconnoitre the spatial signal learning and memory of mice. The average of each group of the two trials that held in the same day was recorded and taken. On the first day, rat in all groups took the full 60 s fixed for the test to attain the platform. From the second day to the fifth day, normal rats and rats feeding on balanced diet containing both sourdough bread samples began to reveal considerable amelioration in comparison to diabetic control rats.

### Impact of diabetes and sourdough bread samples on the time spent in the target quadrant during the probe test

**Fig. 3:**
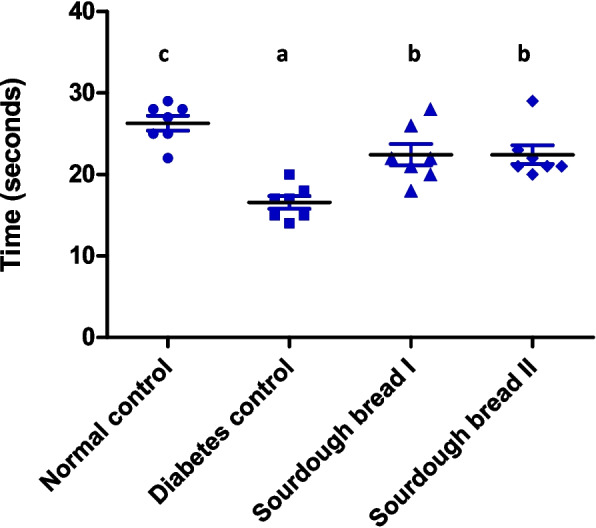
Morris water maze of normal and diabetic groups. Similar letters mean non-significant difference within groups at *P*≤0.05

The average time consumed in the target quadrant for rats feeding on balanced diet containing sourdough bread I and II increased by 6 seconds in comparison to that noted in the group of diabetic control (Fig. 3). However, it was yet considerably shorter than that relate to the normal group.

### The Novel object recognition test

**Fig. 4:**
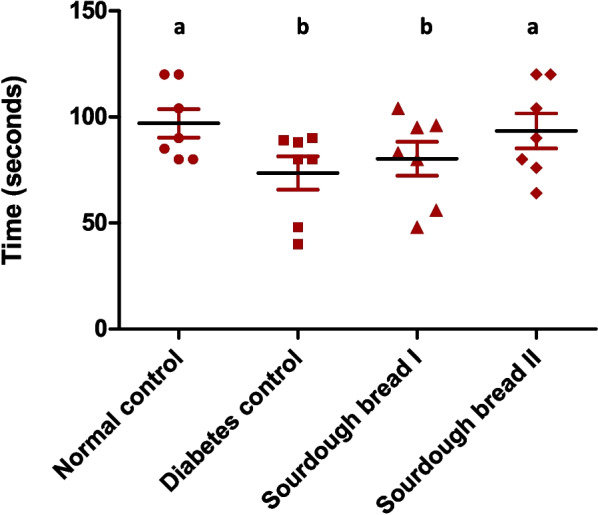
Time spent with new object recognition of normal and diabetic groups. Similar letters mean non-significant difference within groups at *P*≤0.05

NOR test investigates non-spatial memory (long-term remembrance and cognition). The period diabetic control rats, as well as rats feeding on balanced diet containing sourdough bread I spent exploring the new object was significantly lower from the time used by normal rats discovering the novel object (Fig. 4), while rats feeding on balanced diet containing sourdough bread II; spent considerably shorter time in discovering the new object from the time consumed by normal group.

## Discussion

In the present research the impact of diabetes which induced in male rats through injection with Streptozotocin-Nicotinamide (STZ-NC) on biochemical parameters, gene expression and cognitive functions were evaluated. In the present study male rats were used in the model of diabetes due to their sensitivity to cognitive dysfunction as risk factor of diabetes [39].

Our results revealed that insulin was reduced in association with elevation of glucose levels, which proven the induction of type-2 diabetes in rats (Table 4). Reduction of insulin will cause hyperglycaemia [40]. Insulin is responsible for regulation of enzymes that metabolized carbohydrate to maintain blood glucose level. Hyperglycemia is associated with the impairment in insulin excretion with extreme hepatic glycogenolysis and gluconeogenesis; and reduction uptake of glucose by the tissues [41]. Feeding rats on diet containing sourdough bread I or II showed significant elevation of insulin and reduction of glucose, which proven the anti-diabetic effect of the prepared fermented sourdough bread. It was reported previously that sourdough fermentation has been shown reduction in glycaemic index of baked products and enhances phytochemical content and increases the availability of minerals through reduction of anti-nutritional factors [17].

It was reported previously a considerable correlation between diabetes complication and elevation of blood glucose. Hyperglycaemia induces oxidative stress through imbalance between free radicals and antioxidant defence [42]. So, lipid peroxidation (MDA) is an indicator of oxidative stress, which measured in the current research. In models of diabetes in animals, MDA plasma level and catalase activity increased and decreased meaningfully due to hyperglycaemia and oxidative stress induction as shown in the current research. The present results proved that CRP and TNF-α as inflammatory makers elevated significantly in diabetic rats. Diabetic rats feeding on balanced diet containing fermented bakery product I or II exhibited significant reduction in CRP and TNF-α as inflammatory makers with variable degrees. Diabetes mellitus is considered as inflammatory disease due to presence of cytokines such as interleukin (IL)-6 and TNF-α which rose in the blood of diabetic patients [43].

Hyperglycemia induced neuronal and axon injury through rose of oxidative stress in the nervous system [44]. Natural or chemical drugs have improvement effects on histopathology and renal function markers. Renal dysfunction is one of diabetes complication. So any treatment reduce hyperglycemia may be effective in reducing renal dysfunction [45].

The present study indicated that feeding rats on sourdough bread I or II showed significant improvement of plasma lipid profile (Table 5). Fermentation of food materials is a biochemical operation by microorganisms, which presenting different organoleptic characteristic and elevates nutritional value. Consuming fermented foods is linked, with a broad range of dietary supplements beneficial effect such as antioxidant, neuroprotective, anti-inflammatory, immune-modulatory, and hypocholesterolemic properties [46].

Brain-derived neurotrophic factor (BDNF) supports the survival and differentiation of embryonic neurons and controls various neural processes in development and adulthood, including growth, differentiation, survival, synaptic formation, function, and plasticity [47, 48]. Gene expression of BDNF alteration is contributed to many disorder and diseases such as epilepsy, depression, Parkinson and Alzheimer [49–53]. BDNF plays a role in glucose metabolism and may play pathogenetic roles in type-2 diabetes [54]. BDNF ameliorate of hyperglycemia by improving hepatic insulin resistance in diabetes models in animal [55]. Nuclear factor erythroid 2-related factor 2 (Nrf2) regulates anti-inflammatory and antioxidant responses [56, 57]. Nrf2 regulates BDNF transcriptions [58]. Activation of BDNF by transcription factor Nrf2 contributes to antidepressant-like actions in rodents [59, 60]. Studies suggested that lack of Nrf2 may impair both type I and II diabetes [61, 62]. Nrf2 deficient animals showed deteriorated diabetic symptoms and complications in many *in vivo* models [63]. Nrf2 activation ameliorate insulin sensitivity in diabetic patients by suppression of oxidative stress, decrease expression of genes related to gluconeogenesis, reduced weight gain and raise skeletal muscle oxygen exhaustion, and ATP output, as well as increase cellular glucose uptake [9].

GLUT2 is an insulin-independent trans-membrane carrier protein which facilitated glucose movement across cell membranes [64]. PEPCK also called PCK1 is the first rate-limiting enzyme of gluconeogenesis (catalyses the conversion of oxaloacetate to phosphoenolpyruvate) [65]. Up-regulation of GLUT2 and PEPCK expression increased the hepatic glucose production and leads to hyperglycaemia and development of diabetes [66]. The activation of Nrf2 decreases the level of expression of GLUT2 and reactive oxygen species [67]. Overexpression of PEPCK revoked the expression of the nuclear translocation of Nrf2 [68]. In our study; fermented bakery products I and II increased the levels of Nrf2 and BDNF and inhibited glucose production by decreasing the levels of GLUT2 and PEPCK gene expression.

Fermentation of food materials is a biochemical operation by microorganisms, which presenting different organoleptic characteristic and elevates nutritional value [69]. Fermented foods are now considered as “super-foods” for their functional health-enhancer activities [46]. The valuable effects of fermented foods for brain and cognitive function was reported previously [70–73].

The compounds resulted by fermentation of food boost their neuroprotective effects by raising their bioavailability by intestinal absorption and exploitation of the consumed nutrients within the body [74, 75] . Short-chain fatty acids (SCFA) positively effectiveness the host metabolism and play essential role in the CNS [76]. Microbiota may be contributing in the management of absorption of phytochemicals and improves their activities as antioxidant and anti-inflammatory [77, 78]. Probobiotic supplementation possessed alleviation in memory dysfunction and neuroinflammation through their activities as anti-inflammatory and antioxidant; via lipopolysaccharides-induced suppression of acetylcholeinesterase [79].

Nutritional intervention is the best efficient way to moderating the gut microbiota. Dietary supplements influence the gut microbial composition and directly affect neural functioning in both the ENS and CNS [80, 81]. Diet containing fruits and vegetables as source of antioxidants, probiotics, flaxseed, nuts and fish as source of omega-3 polyunsaturated fatty acids has been shown to alleviate inflammation of the neuron and reduce the chance of cognitive dysfunction and finally AD [82, 83].

In the present study sourdough bread samples I and II containing fibers as shown from the proximate analysis (Table 3), polyunsaturated fatty acids (Table 3) and phenolic compounds (Table 3). Fibers, phenolic compounds and polyunsaturated fatty acids have prebiotic properties and play an important role in improving cognitive performance in animal models through influence on biochemical pathways such as brain derived neurotropic factor [18, 19]. Linolenic acid (ALA) (C18:3, ω-3), which is present in both prepared bread samples (Table 3); when consumed is metabolized to EPA (20:5 ω-3) and DHA (22:6 ω-3) by series of desaturation and elongation reactions. EPA (20:5 ω-3) and DHA (22:6 ω-3) are essential PUFA and are important component of cell membranes. Both EPA and DHA reportedly have neuroprotective effects via mechanisms such as suppression of inflammation, regulation of neurogenesis, and protection against oxidative stress [84]. Omega-3 fatty acids significantly induce neovasculogenesis in high glucose-mediated endothelial progenitor cells in type-2 diabetes. Also they are effective in diabetic retinopathy via suppression of angiogenesis [85].

## Conclusion

The outcomes of the present research could support the WHO guidelines about the effect of diabetes on increasing cognitive dysfunction incidence. The studied sourdough breads samples were effectively improved diabetes status in diabetic rats and also reduced the retardation in cognitive function due to diabetes. Sourdough bread II was superior to Sourdough bread I. The anti-diabetic effect and protection from cognitive dysfunction of sourdough bread samples may be ascribed to the occurrence of dietary fibers, phenolic compounds, and polyunsaturated fatty acids.

## Data Availability

The data that support the findings of this study are available from corresponding author but restrictions apply to the availability of these data, which were used under license for the current study, and so are not publicly available. Data are however available from the authors upon reasonable request and with permission of corresponding author. All the relevant data found in the study are available in the article.

## Abbreviations

AD: Alzheimer’s disease
ANOVA: Analysis of variance
BDNF: Brain-derived neurotrophic factor
CRP: C-reactive protein
GLUT2: Glucose transporter 2
MDA: Malondialdehyde
NRF-2: Nuclear respiratory factor 2
PEPCK: Phosphoenol pyruvate carboxy kinase
STZ-NC: Streptozotocin-Nicotinamide
T2D: Type2-diabetes
